# Mild autonomic dysfunction in primary Sjögren's syndrome: a controlled study

**DOI:** 10.1186/ar2385

**Published:** 2008-03-07

**Authors:** Fin ZJ Cai, Sue Lester, Tim Lu, Helen Keen, Karyn Boundy, Susanna M Proudman, Anne Tonkin, Maureen Rischmueller

**Affiliations:** 1 Rheumatology Department, The Queen Elizabeth Hospital, Woodville Road, Woodville South, 5011, Australia; 2 Hanson Institute, Frome Road, Adelaide, 5000, Australia; 3 Neurology Department, The Queen Elizabeth Hospital, Woodville Road, Woodville South, 5011, Australia; 4 Rheumatology Department, The Royal Adelaide Hospital, North Terrace, Adelaide, 5000, Australia; 5 School of Medicine, University of Adelaide, Frome Road, Adelaide, 5000, Australia

## Abstract

**Introduction:**

The aim of this study was to compare cardiovascular autonomic nervous system function in patients with primary Sjögren's syndrome (pSS) with that in control individuals, and to correlate the findings with autonomic symptoms and the presence of exocrine secretory dysfunction.

**Methods:**

Twenty-seven female patients with pSS and 25 control individuals completed the COMPASS (Composite Autonomic Symptom Scale) self-reported autonomic symptom questionnaire. Beat-to-beat heart rate and blood pressure data in response to five standard cardiovascular reflex tests were digitally recorded using a noninvasive finger pressure cuff and heart rate variability was analyzed by Fourier spectral analysis. Analysis was performed by analysis of variance (ANOVA), multivariate ANOVA and repeated measures ANOVA, as indicated. Factor analysis was utilized to detect relationships between positive autonomic symptoms in pSS patients.

**Results:**

Multiple, mild autonomic disturbances were observed in pSS patients relating to decreased heart rate variability, decreased blood pressure variability and increased heart rate, which were most evident in response to postural change. There was a strong trend toward an association between decreased heart rate variability and increased severity of the secretomotor, orthostatic, bladder, gastroparesis and constipation self-reported autonomic symptom cluster identified in pSS patients. This symptom cluster was also associated with fatigue and reduced unstimulated salivary flow, and therefore may be an important component of the clinical spectrum of this disease.

**Conclusion:**

There was evidence of mild autonomic dysfunction in pSS as measured with both cardiovascular reflex testing and self-reported symptoms. Pathogenic autoantibodies targeting M3 muscarinic receptors remain a strong candidate for the underlying pathophysiology, but practical assays for the detection of this autoantibody remain elusive.

## Introduction

Primary Sjögren's syndrome (pSS) is a systemic autoimmune disease that is characterized by exocrine failure of salivary and lacrimal glands, in addition to a wide range of extraglandular features. Many clinical features of pSS are also features of autonomic neuropathy, which has been documented in pSS [1]. Functional autoantibodies that target muscarinic acetylcholine receptors have been identified in the sera of patients with pSS, and these may represent an important mechanism in the production of sicca symptoms, bladder irritability and gastrointestinal symptoms [2,3]. Cardiovascular autonomic responses are a validated measure of autonomic nervous system function, and analysis of heart rate variability (HRV) provides additional information about parasympathetic and sympathetic activity. Studies in this area have yielded variable results in pSS patients, depending on the population studied and methodology applied. Some reported no autonomic dysfunction [4,5] whereas others found disturbance of the parasympathetic system [6,7] or both parasympathetic and sympathetic nervous systems [8-10]. The aim of this study was to compare objectively autonomic nervous system function in pSS patients with that in control individuals, and to correlate the findings with objective measures of secretomotor function and self-reported symptoms.

## Materials and methods

### Study participants

Female pSS patients were recruited consecutively from the Rheumatology Clinic at The Queen Elizabeth Hospital. All patients met the revised 2002 American European Consensus criteria [11] for pSS. Age-matched, population-based female control individuals were recruited from the local community. Exclusion criteria for the study included diabetes, ischaemic heart disease, current anticholinergic medication, or a serious medical illness. Seven participants from each group were taking antihypertensive medications, which were withheld for 24 hours before testing. Classes of medications used by pSS patients and control individuals (respectively) were as follows: angiotensin-converting enzyme inhibitors (one and two participants), β-blockers (one and two participants), angiotensin receptor blockers (three and four participants), diuretics (two and three participants), calcium channel blockers (three and no participants) and hydrallazine (one and no participants). Five pSS patients were using pilocarpine, which was withheld for 24 hours before testing. Two control individuals in whom cardiac arrhythmias were detected during cardiovascular reflex testing were excluded from the analysis, and 27 pSS patients and 25 control individuals were included in the final study.

Eighteen (67%) of the pSS patient sera were positive for Ro/La autoantibodies, and of those tested nine out of nine (100%) were negative for cryoglobulins and two out of 17 (12%) had low C3 or C4 levels. Eleven patients (41%) had Raynaud's phenomenon, and of those tested 13 out of 14 (93%) had a positive labial salivary gland biopsy. The average age of onset of disease was 48 years (range 29 to 73 years) and the average disease duration was 13 years (range 2 to 29 years).

All participants gave informed, written consent for the study, and the study was approved by the North Western Adelaide Health Service Ethics of Human Research Committee.

### Study protocol

All testing was conducted in the morning and in a standardized manner. Hypertensive medications were withheld 24 hours before testing, participants abstained from caffeine and cigarettes from the previous evening, and artificial tears from waking that morning. Patients were instructed to have an early light breakfast, and testing did not commence until the patients had been fasting for more than 1 hour.

Participants initially completed the FACIT-F (Functional Assessment of Chronic Illness Therapy-Fatigue), a 13-item assessment of fatigue [12], and the COMPASS (Composite Autonomic Symptom Scale) questionnaire [13]. A 15-minute unstimulated whole salivary flow and Schirmer's-I test were performed as objective measures of dryness. Sicca was defined as an unstimulated salivary flow test of under 1.5 ml in 15 minutes and/or a Schirmer's test with under 5 mm wetting in both eyes over 5 minutes.

Physical activity levels were measured using the short telephone form of the International Physical Activity Questionnaire [14]. Participants were classified as HEPA (health enhancing physical activity) active if they achieved either of the following: activity of vigorous intensity on at least 3 days, achieving a minimum of at least 1,500 multiples of resting metabolic state (MET)-minutes/week; or 7 or more days of any combination of walking, or activity of moderate intensity or vigorous intensity achieving a minimum of at least 3,000 MET-minutes/week. All study participants were examined by a neurologist. Abnormalities were observed in five pSS patients: bilateral carpal tunnel syndrome (in one patient), an old minor cerebrovascular accident (in one), unilateral benign essential tremor (in one), peripheral neuropathy (in two) and facial numbness (in one).

### Cardiovascular reflex testing

Noninvasive, beat-to-beat measurements of systolic blood pressure (SBP), diastolic blood pressure, mean blood pressure (MBP), heart rate and heart period (RR interval) were recorded during all manoeuvres using the Finapres™ (Ohmeda. Louisville, Colorado, USA) finger arterial pressure monitoring system [15].

Manoeuvres (described below) were performed in the following order: supine rest, postural change, Valsalva manoeuvre (seated), isometric grip (seated) and controlled breathing (seated). Participants rested for several minutes between successive manoeuvres and between replicates of manoeuvres.

#### Supine rest

Participants lay quietly on a bed, and once settled recording commenced for a period of 5 minutes. Brachial blood pressure was measured using a digital blood pressure monitor approximately 1 minute before completion of this period.

#### Postural change

Participants were asked to stand quickly and remain standing quietly for a period of 6 minutes. Brachial blood pressure was measured with a digital blood pressure monitor at 2 and 5 minutes after standing.

#### Valsalva manoeuvre (seated)

Participants blew into a closed tube with a small leak, maintaining an expiratory pressure of 40 mmHg for 10 seconds. Beat-to-beat measurements were monitored for a period of 1 minute after release of expiratory pressure. This manoeuvre was performed three times.

#### Isometric grip (seated)

Patients gripped a dynamometer for 3 minutes, maintaining a contraction pressure of one-third of their maximum voluntary contraction pressure.

#### Controlled breathing (seated)

Participants maintained a controlled, even breathing rate of six breaths/minute over a period of 1 minute. This manoeuvre was performed three times.

### Cardiovascular reflex test analysis

Five standard parameters of the cardiovascular autonomic test were estimated [16]: supine to standing ΔSBP, supine to standing 30/15 ratio, isometric grip ΔMBP, Valsalva ratio, and breathing E/I ratio.

#### Supine to standing ΔSBP

The supine to standing ΔSBP was calculated as the brachial SBP 5 minutes after standing minus the supine brachial SBP.

#### Supine to standing 30/15 ratio

The supine to standing 30/15 ratio is the ratio of the longest RR interval near to the 30th beat after standing to the shortest RR interval near to the 15th beat after standing.

#### Isometric grip ΔMBP

The isometric grip ΔMBP is the MBP (Finapres™; Ohmeda) at the end of the 3-minute grip period minus the MBP just before commencing grip.

#### Valsalva ratio

The Valsalva ratio is the ratio of the longest RR interval immediately after strain to the shortest RR interval during strain. The geometric mean was estimated from three replicates.

#### Breathing E/I ratio

The breathing E/I ratio is the mean of the longest RR intervals during each expiration divided by the mean of the shortest RR intervals during each inspiration [17]. The geometric mean was estimated from three replicates.

### Heart rate variability analysis

#### Time domain analysis

The mean and time domain HRV parameters of the RR interval [18], including the standard deviation (SDNN), the proportion of successive intervals differing by more than 50 ms (pNN50) and the standard deviation of the differenced RR series (RMSSD), were calculated for both supine and standing (beginning 1 minute after standing) positions over a 5-minute recording interval.

#### Frequency domain (spectral) analysis

Cross-spectral analysis of the beat-to-beat RR and SBP data was performed on both supine and standing 5-minute recording intervals. The data were interpolated at a frequency of 2 Hz using cubic spline interpolation. Exact length cross-spectral Fourier analysis was performed using the Time Series module of Statistica (v6.1; Statsoft Inc., Tulsa, Oklahoma, USA), with a taper of 15% and a Hamming window of width five to estimate the spectral densities. Power was calculated by integration of the spectral densities over the frequency ranges of 0.04 to 0.15 Hz (low frequency [LF]) and 0.15 to 0.4 Hz (high frequency). The gain, essentially a regression coefficient for the SBP variability as a predictor of RR variability, was used as a measure of baroreflex function [19]. This was estimated as the total cross-amplitude power divided by the total SBP power over the relevant frequency range.

### Statistical analysis

The cardiovascular reflex test scores were analyzed as continuous variables rather than classified as normal, borderline and abnormal, as initially described [16]. This is because there was a substantial age dependence in these scores, also recognized in other studies [20], that is not incorporated into the classification criteria. All analyses were performed by analysis of variance (ANOVA), multivariate ANOVA and repeated measures ANOVA, as indicated. With the exception of blood pressure, measurements of autonomic function and HRV, which were either ratios or rate measurements, were log-transformed before analysis to normalize their distribution. Reported results are for an age-unadjusted analysis, but they did not differ from results for an age-adjusted analysis. Many of the cardiovascular reflex test and COMPASS domain scores were highly correlated with each other. Therefore principal component factor analysis was employed to detect structure in the relationships between parameters. All factors with a minimum Eigan value of 1 were extracted. All analyses were performed using Statistica (v6.1; Statsoft Inc.).

## Results

### Baseline characteristics

All study participants were female and their baseline characteristics are shown in Table 1. pSS patients and control individuals were well matched in terms of age. Physical activity level, SBP, hypertension and prior smoking history were similar between groups. As expected, there was a higher incidence of objective sicca symptoms in pSS patients, measured just before autonomic testing, and more severe fatigue.

**Table 1:** Baseline characteristics of pSS patients and control individuals

	pSS patients	Control individuals	*P*
Number of participants (all female)	27	25	
Age (years; range)	60 (40–79)	60 (42–79)	0.98
Supine brachial SBP (mmHg [95% confidence interval])	131 (124 to 138)	134 (127 to 141)	0.53
Hypertension diagnosis (*n*/*n *[%])	7/27 (26%)	10/25 (40%)	0.43
Prior smokers^a ^(*n*/*n *[%])	9/27 (33%)	10/25 (40%)	0.83
HEPA active^b ^(*n*/*n *[%])	8/25 (32%)	9/25 (36%)	1.0
FACIT-F score (standard error)	20.6 (2.6)	10.0 (2.0)	0.002
Sicca^c ^(*n*/*n *[%])	22/26 (85%)	5/25 (20%)	0.00001

^a^None of the participants were current smokers. ^b^HEPA (Health Enhancing Physical Activity) active is the highest physical activity level, as measured using the International Physical Activity Questionnaire. ^c^Sicca was defined as an unstimulated salivary flow test of under 1.5 ml in 15 minutes and/or a Schirmer's test with under 5 mm wetting in both eyes. FACIT-F, Functional Assessment of Chronic Illness Therapy-Fatigue; pSS, primary Sjögren's syndrome; SBP, systolic blood pressure.

### COMPASS scores

Self-reported autonomic symptoms, as assessed using the COMPASS score (Table 2), were increased in pSS patient relative to control individuals (34.2 versus 15.3; *P *= 0.0002). These scores are consistent with normal autonomic function in control individuals and mild to moderate symptom severity in pSS patients when interpreted against COMPASS validation scores of 9.8 (± 9) for control individuals, 25.9 (± 17.9) for patients with nonautonomic peripheral neuropathy, and 52.3 (± 24.2) for patients with autonomic failure [13]. The understatement scores were modest and comparable between pSS patients and control individuals. pSS patients scored higher in the psychosomatic component (mean 0.60 versus 0 out of a maximum score of 10; *P *= 0.006; Table 2). However, these scores were in fact low and primarily attributable to pSS patients reporting difficulty in swallowing, which is a component of the COMPASS psychosomatic score but also a common symptom of pSS associated with dry mouth.

**Table 2:** COMPASS scores in pSS patients versus control individuals

		Mean (95% CI)		
			
COMPASS score	Maximum score	pSS patients	Control individuals	*P*
Subscale				

Orthostatic intolerance	40	10.2 (7.4 to 13.9)	4.5 (2.1 to 9.4)	0.029*
Bladder disorder	20	4.7 (3.5 to 6.3)	1.8 (0.8 to 4.0)	0.007*
Diarrhoea	20	3.0 (1.9 to 4.7)	1.1 (0.3 to 4.0)	0.07
Gastroparesis	10	1.2 (0.8 to 2.0)	0.5 (0.1 to 1.7)	0.08
Secretomotor disorder	20	6.8 (5.8 to 8.1)	2.4 (1.5 to 3.9)	0.000002*
Sleep disorder	15	2.2 (1.6 to 3.0)	1.5 (0.9 to 2.5)	0.23
Constipation	10	0.7 (0.3 to 1.7)	1.4 (0.9 to 2.2)	0.10
Vasomotor	10	2.9 (2.1 to 4.0)	0.6 (0.1 to 3.1)	0.002*
Pupillomotor impairment	5	1.9 (1.5 to 2.3)	1.2 (0.9 to 1.7)	0.02*
Syncope	20	0.6 (0.3 to 1.2)	0.2 (0.1 to 2.7)	0.18

COMPASS Total	170	34.2 (28.2 to 41.3)	15.3 (9.8 to 23.8)	0.0002*

Understatement	10	2.28 (1.22 to 3.35)	2.56 (1.57 to 3.55)	0.70
Psychosomatic	10	0.60 (0.24 to 1.06)	0	0.006*

*Statistically significant finding (*P *< 0.05). CI, confidence interval; COMPASS, Composite Autonomic Symptom Scale; pSS, primary Sjögren's syndrome.

When analyzed by symptom subscale (Table 2), the most substantive difference between pSS patients and control individuals was the secretomotor subscale scores, as expected. There was also evidence of bladder dysfunction, as we previously reported [21], in addition to orthostatic intolerance, and vasomotor and pupillomotor dysfunction.

**Figure 1:**
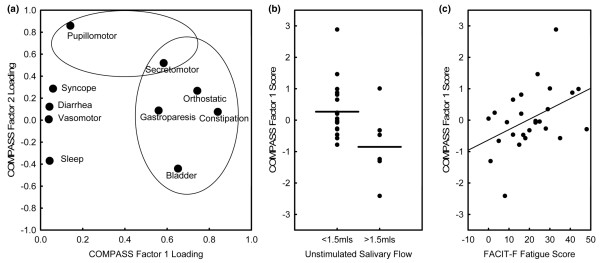
Factor analysis of COMPASS autonomic symptom scale scores within pSS patients. **(a) **Scatterplot of rotated (varimax normalized) COMPASS subscale factor loadings for factor 1 (24% of total variance) and factor 2 (15% of total variance), both with appreciable loadings for the secretomotor subscale. Factor 1 had the highest loadings for secretomotor, orthostatic, gastroparesis, constipation and bladder subscales, which is indicative of a substantial clustering of these symptoms within patients who have primary Sjögren's syndrome (pSS). The highest loadings for factor 2 were observed with both the secretomotor and pupillomotor subscales. **(b) **Scatterplot of the COMPASS factor 1 scores for each pSS patient by results of the contemporaneous 15-minute unstimulated salivary flow test. The horizontal bars represent mean scores for each group. Factor 1 scores were significantly higher in patients with this objective measure of dryness (*P *= 0.025), whereas factor 2 scores were not (*P *= 0.40; data not shown). Scatterplot of the COMPASS factor 1 scores for each pSS patient by the FACIT-F scores. Factor 1 scores were significantly correlated with fatigue scores (*P *= 0.035), whereas factor 2 scores were not (*P *= 0.18; data not shown). COMPASS, Composite Autonomic Symptom Scale; FACIT-F, Functional Assessment of Chronic Illness Therapy-Fatigue.

There were multiple correlations between the COMPASS symptom subscales, and factor analysis was employed to analyze clustering of symptoms within pSS patients. Within pSS patients, four independent factors were extracted that accounted for 73% of the total variance. The secretomotor subscale had substantial factor loadings for both factor 1 (24% variance), for which additional high loadings were observed for orthostatic, bladder, constipation and gastroparesis subscales; and factor 2 (15% variance), which had an additional high loading on the pupillomotor subscale (Figure 1a). Factor 3 (22% variance) had substantial loadings on vasomotor, gastroparesis and syncope subscales, whereas factor 4 (12% variance) had substantial negative loadings on diarrhoea and sleep subscales. Importantly, factor 1 scores for each patient were associated with both objective sicca, as measured by 15-minute unstimulated salivary flow (*P *= 0.025, Figure 1b), and the FACIT-F scores (Spearman rank correlation coefficient 0.42; *P *= 0.035; Figure 1c). Therefore, autonomic dysfunction is a component of pSS and manifests in symptoms additional to secretory dysfunction. There were no associations (*P *= 0.40 and *P *= 0.18, respectively) with factor 2 scores, which may be interpreted as measures of parasympathetic function. Furthermore, there were no associations with Ro/La autoantibody status or Raynaud's phenomenon.

### Standard cardiovascular autonomic tests

There were significant differences between pSS patients and control individuals in the five standard measures of cardiovascular autonomic testing (multivariate *P *= 0.018, Table 3). Individually significant differences were specifically related to postural change. There was both an attenuated increase in brachial ΔSBP (*P *= 0.031) and an attenuation in the RR 30/15 ratio (*P *= 0.001) in response to standing in pSS patients. There was no evidence of any differences in the Valsalva ratio, MBP response to isometric grip, or E/I ratio during controlled breathing.

**Table 3:** Cardiovascular autonomic tests in pSS patients versus control individuals

	Mean (95% CI)		
		
Cardiovascular autonomic test	pSS patients	Control individuals	*P*
Supine to standing: ΔSBP	+2 (-2 to +6)	9 (4 to 14)	0.031*
Supine to standing: 30/15 ratio	1.19 (1.14 to 1.24)	1.33 (1.27 to 1.40)	0.001*
Isometric grip: ΔMBP	22 (17 to 27)	27 (3 to 21)	0.24
Valsalva ratio	1.25 (1.18 to 1.33)	1.31 (1.25 to 1.37)	0.23
Breathing E/I ratio	1.17 (1.14 to 1.20)	1.18 (1.15 to 1.21)	0.66
Multivariate *P *value = 0.018			

CI, confidence interval; ΔMBP, change in Finapres™ mean blood pressure (end of grip minus before grip); ΔSBP, change in brachial systolic blood pressure (5 minutes standing minus supine); pSS, primary Sjögren's syndrome.

**Figure 2:**
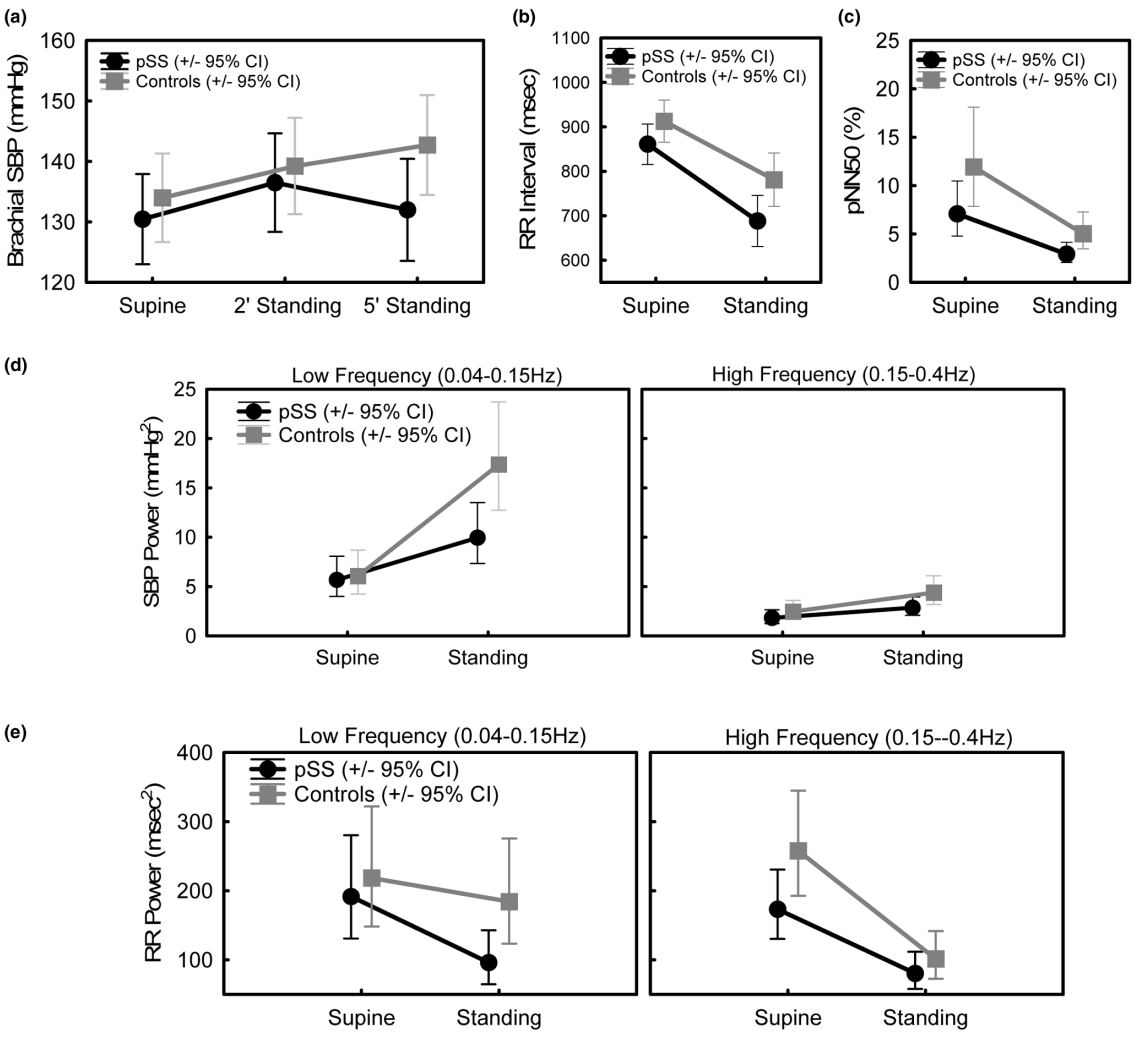
Abnormal HRV responses after postural change in pSS patients. All analyses were performed by repeated measures analysis of variance. **(a) **Brachial systolic blood pressure (SBP). The initial SBP response to standing was normal in patients who have primary Sjögren's syndrome (pSS). However, between 2 and 5 minutes after standing, there was a relative decline in SBP in pSS patients and a relative increase in control individuals (*P *= 0.015). **(b) **RR intervals. There was a relative tachycardia in pSS patients. This was most pronounced during standing (*P *= 0.039). **(c) **The proportion of successive RR intervals differing by more than 50 ms (pNN50) was lower in pSS patients than in control individuals over both postural positions (*P *= 0.025). **(d) **SBP power. The normal response to standing is an increase in SBP power, most evident in the low frequency (0.04 to 0.15 Hz) domain. This was significantly attenuated in pSS patients (*P *= 0.01). **(e) **RR power. Parasympathetic withdrawal upon standing results in a decrease in heart rate variability (HRV). In the low frequency (LF) domain, this is counterbalanced by an increase associated with increased LF blood pressure variability (see panel c). The net result of a normal response to standing is very little change in LF HRV and a substantial decrease in high frequency HRV. In control individuals, there was minimal change in LF HRV in response to standing, consistent with a normal response. However, in pSS there was a substantial decrease in LF HRV upon standing, and therefore standing LF HRV was significantly lower in pSS patients (*P *= 0.024).

The brachial SBP response to standing was further analyzed at both 2 and 5 minutes after standing. The difference in ΔSBP (Table 3) between pSS patients and control individuals can be traced to a decline in SBP between 2 and 5 minutes standing in pSS patients, as compared with a relative increase in the same time period in control individuals (Figure 2a). Two pSS patients had to be seated before completion of the standing exercise (and were therefore excluded from this component of the analysis) because they exhibited symptoms of postural hypotension such as dizziness and nausea associated with a decline in blood pressure.

Although the observed differences in the ΔSBP and standing RR 30/15 ratio between pSS patients and control individuals are consistent with some orthostatic intolerance in pSS, also observed in the COMPASS subscale scores (Table 2), the magnitude of these differences is relatively modest and the values are within the normal range [16]. This suggests the presence of mild, possibly subclinical autonomic dysfunction in pSS.

### Heart rate variability: time domain measures

There was a relative tachycardia in pSS patients (Figure 2b) as assessed by repeated measures ANOVA for both supine and standing positions. This relative tachycardia was most pronounced during standing (*P *= 0.039), but there was no evidence that the decrease in RR intervals associated with postural change was different between patients and control individuals (*P *= 0.21, by repeated measures ANOVA). The mean standing RR intervals in pSS patients was 688 ms (95% confidence interval 670 ms to 755 ms), as compared with 781 ms (95% confidence interval 735 ms to 828 ms) in control individuals.

Standard time domain estimates of HRV include SDNN, RMSSD and pNN50 [18]. There was a trend toward decreased HRV in pSS relative to control individuals in all three measures, but only the pNN50 frequency was significant (*P *= 0.025; Figure 2c). There was no evidence that the decrease in pNN50 associated with postural change was different between patients and control individuals (*P *= 0.94, repeated measures ANOVA).

### Heart rate variability: spectral analysis

There were differences in the spectral (or power) analysis between pSS patients and control individuals, predominantly in the LF power range, in response to standing. The normal SBP variability response to standing is an increase in LF power. This was significantly attenuated in pSS patients (*P *= 0.01; Figure 2d). Parasympathetic withdrawal upon standing results in a decrease in HRV. In the LF domain this is counterbalanced by increased LF blood pressure variability, and the net result of a normal response to standing is little change in LF HRV. In control individuals there was minimal change in LF HRV in response to standing, which is consistent with a normal response. However, in pSS there was a substantial decrease in LF HRV (*P *= 0.024; Figure 2e). There were no differences in the baroreflex function, as estimated by the cross-spectral LF gain (data not shown).

### Factor analysis of cardiovascular autonomic indices

**Figure 3:**
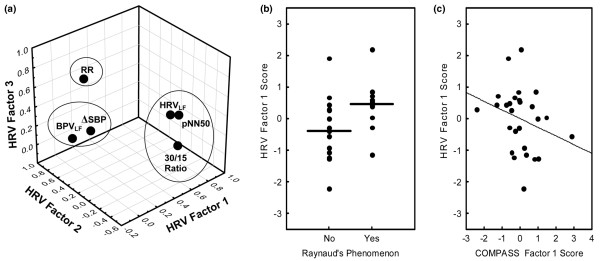
Factor analysis of abnormal postural change cardiovascular autonomic indices within pSS patients. RR intervals, low frequency RR power (HRV_LF_), proportion of successive RR intervals differing by more than 50 ms (pNN50), change in systolic blood pressure on standing (ΔSBP), low frequency systolic blood pressure power (BPV_LF_) and the 30/15 ratio were all decreased in patients who have primary Sjögren's syndrome (pSS) relative to control individuals on standing. **(a) **Three-dimensional scatterplot of rotated (varimax normalized) factor loadings. Factor 1 had the highest loadings for HRV_LF_, pNN50 and the 30/15 RR ratio. Factor 2 had the highest loadings for ΔSBP and BPV_LF_. Factor 3 had the highest loading for RR intervals during standing. **(b) **Scatterplot of the HRV factor 1 scores for each pSS patient by the presence of Raynaud's phenomenon. The horizontal bars represent mean scores for each group. Factor 1 scores were significantly lower (more abnormal) in patients without Raynaud's (*P *= 0.025). **(c) **Scatterplot of the HRV factor 1 scores (y-axis) versus COMPASS factor 1 scores (x-axis) for each pSS patient. There was a negative correlation that did not quite reach statistical significance (*P *= 0.08).

RR intervals, LF RR power (HRV_LF_), pNN50, change in SBP on standing (ΔSBP), LF SBP power and the 30/15 ratio were all decreased in pSS patients relative to control individuals on standing. Because there were multiple correlations between these indices, factor analysis was again employed for pSS patient data to detect clustering or structural relationships between these indices and enhance interpretation. Three independent factors (Figure 3a) were extracted, which accounted for 75% of the total variance, and this is indicative of multiple autonomic abnormalities in pSS patients. Factor 1 (33% variance) had the highest loadings for HRV_LF_, pNN50 and the 30/15 RR ratio, and may be interpreted as a HRV factor, possibly reflecting sympathetic/parasympathetic balance. Interestingly, factor 1 scores were higher (less abnormal) in patients with Raynaud's phenomenon (*P *= 0.025; Figure 3b) which is associated with sympathetic overactivity [22]. Furthermore, there was a modest correlation with the COMPASS autonomic symptom factor 1, which did not quite reach statistical significance (*P *= 0.08; Figure 3c). Factor 2 (22% variance) had the highest loadings for blood pressure variability (ΔSBP and LF SBP power). Factor 3 (20% variance) had the highest loading for heart rate (RR intervals). There was no relationship between these cardiovascular factors and Ro/La autoantibodies, objective sicca measures, or fatigue scores.

## Discussion

In this study we demonstrated evidence, obtained from both self-reported symptoms and objective cardiovascular reflex testing, of mild autonomic dysfunction in pSS. From the cardiovascular reflex testing, there was evidence of multiple autonomic disturbances in pSS relating to decreased HRV, decreased blood pressure variability and an increased heart rate (tachycardia), which were most evident in response to postural change. There was a strong trend toward an association between decreased HRV and increased severity of the secretomotor, orthostatic, bladder, gastroparesis and constipation self-reported symptom cluster in pSS patients. This symptom cluster was also associated with fatigue and reduced unstimulated salivary flow, and therefore may be an important component of the clinical spectrum of this disease. Of note, we [21] and others [23] previously reported increased bladder symptoms in pSS patients; furthermore, a high frequency of both delayed gastric emptying and decreased bladder detrusor muscle tone has also been observed in pSS patients [24].

Previous studies addressing cardiovascular autonomic function in pSS have yielded conflicting results, although a pattern is emerging. Two studies using 24-hour Holter monitoring [4,5], which reflects tonic balance, both reported negative results. In contrast, a number of studies of provoked cardiovascular responses or short-term HRV [6-10,25-27] identified abnormalities in pSS, although not all of these were controlled studies or used appropriate age-adjusted criteria for interpretation of abnormal test results. Four controlled studies [8-10,27] found abnormalities in either the 30/15 ratio or blood pressure response to postural challenge, as we also observed, and two controlled studies [6,26] identified reduced HRV/blood pressure variability in pSS patients by using spectral analysis. Our observation of a relative tachycardia in pSS patients has not previously been reported. Four studies [8-10,27] also reported a decreased breathing E/I ratio in pSS patients, which we did not observe. However, this test is also influenced by breathing tidal volume, which may have differed between study participants and potentially confounded the results. Similar to other studies [10], we did not observe an association between cardiovascular reflex test scores and objective measures of sicca in pSS patients, but this is the first study to both examine and report an association between objective measures of sicca and self-reported autonomic symptoms in pSS patients.

Potential mechanisms of autonomic dysfunction in SS include T-cell infiltration and destruction of ganglions and nerves [28], cytokine-induced inhibition of neuropeptide secretion from nerve endings [29], immune complex-mediated inflammation (although few pSS patients in this study exhibited cryoglobulins and/or low C3 or C4, which might indicate immune complex deposition), and pathogenic autoantibodies targeting receptors relevant for autonomic functioning [30]. IgG autoantibodies, which inhibit the function of type 3 muscarinic receptors (M3Rs), have been described in pSS patients [3,31]. Importantly, these autoantibodies inhibit salivary secretion [32], bladder detrusor muscle contraction [3], and colon contractions [33]*in vitro*. Evidence that lower urinary tract symptoms in pSS are autoantibody mediated comes from passive transfer of SS immunoglobulin or rabbit anti-M3R to mice, which produces the phenotype of overactive bladder [2]. Furthermore, neutralization of anti-M3R autoantibodies by intravenous immunoglobulin led to improvement in bladder and bowel autonomic symptoms in patients with autoimmune diseases [34]. Therefore, pathogenic M3R autoantibodies are strongly implicated in the pathophysiology of the cluster of secretomotor, bladder, gastroparesis, constipation and orthostatic autonomic symptoms in pSS patients observed in the present study.

Pathogenic M3R autoantibodies may also potentially influence cardiovascular autonomic responses. Although the M2R subtype is the numerically and functionally predominant muscarinic receptor in the heart, recent studies have provided compelling and solid evidence in support of the important roles of M3R in regulating and maintaining cardiac function and heart disease [35]. Furthermore, given the close structural similarity between the M2R and M3R, it is likely that the autoantibodies may be cross-reactive.

Muscarinic receptor-mediated cardiac parasympathetic activity is essential for regulating heart rate [35] and HRV [36]. Furthermore, vasodilatory responses to cholinergic stimuli are diminished in M3R knockout mice [37] and in pSS patients [38], which may – at least in part – underpin the reduced blood pressure variability observed in the present study. Cardiovascular reflex tests are traditionally interpreted as an indication of parasympathetic or sympathetic function, but our results are better interpreted as multiple autonomic disturbances in pSS relating to decreased HRV, decreased blood pressure variability and increased heart rate, which are likely to reflect a disturbance of parasympathetic/sympathetic balance.

## Conclusion

We have confirmed the presence of mild autonomic dysfunction in pSS patients, as measured by both self-reported symptoms and objective assessment. We have identified an important cluster of self-reported secretomotor, orthostatic, bladder, gastroparesis and constipation symptoms in pSS, which correlate with increased fatigue and reduced serum salivary flow. Cardiovascular reflex testing reveals multiple abnormalities that reflect probable disturbance of parasympathetic/sympathetic balance. Although pathogenic M3R autoantibodies remain a strong candidate for the underlying pathophysiology in pSS, it is not yet possible to test this hypothesis, because practical assays for anti-M3R autoantibody detection remain elusive.

## Abbreviations

ANOVA= analysis of variance; COMPASS = Composite Autonomic Symptom Scale; FACIT-F = Functional Assessment of Chronic Illness Therapy-Fatigue; HRV = heart rate variability; LF = low frequency; M3R = type 3 muscarinic receptor; MBP = mean blood pressure; MET = multiples of resting metabolic state; pNN50 = the proportion of successive RR intervals differing by more than 50 ms; pSS = primary Sjögren's syndrome; RMSSD = standard deviation of the differenced RR interval series; SBP = systolic blood pressure; SDNN = standard deviation of the RR interval series.

## Competing interests

The authors declare that they have no competing interests.

## Authors' contributions

FZC recruited patients, was responsible for data management, and carried out autonomic testing and drafted the manuscript. SL assisted with autonomic testing, performed statistical analysis and assisted with manuscript preparation. TL performed additional patient recruitment and autonomic testing. HK and AT were responsible for test selection and training in autonomic testing. KB assisted with study design and performed neurological examinations. SP assisted with the study design and patient ascertainment. MR conceived of the study, participated in its design and coordination and drafting of the manuscript. All authors read and approved the final manuscript.
